# The triple combination of Remdesivir (GS-441524), Molnupiravir and Ribavirin is highly efficient in inhibiting coronavirus replication in human nasal airway epithelial cell cultures and in a hamster infection model

**DOI:** 10.1016/j.antiviral.2024.105994

**Published:** 2024-09-03

**Authors:** Thuc Nguyen Dan Do, Rana Abdelnabi, Bernadett Boda, Samuel Constant, Johan Neyts, Dirk Jochmans

**Affiliations:** a KU Leuven, Department of Microbiology, Immunology and Transplantation, Rega Institute, Laboratory of Virology and Chemotherapy, 3000, Leuven, Belgium; b The VirusBank Platform, Gaston Geenslaan, B-3000, Leuven, Belgium; c Epithelix Sàrl, 18 Chemin des Aulx, Plan-les-Ouates, CH-1228, Geneva, Switzerland

## Abstract

The use of fixed dose-combinations of antivirals with different mechanisms of action has proven key in the successful treatment of infections with HIV and HCV. For the treatment of infections with SARS-CoV-2 and possible future epi-/pandemic coronaviruses, it will be important to explore the efficacy of combinations of different drugs, in particular to avoid resistance development, such as in patients with immunodeficiencies. This work explores the effect of a combination of 3 broad-spectrum antiviral nucleosides on the replication of coronaviruses. To that end, we made use of primary human airway epithelial cell (HAEC) cultures grown at the air-liquid interface that were infected with the beta coronavirus OC43. We found that the triple combination of GS-441524 (the parent nucleoside of remdesivir), molnupiravir and ribavirin resulted in a more pronounced antiviral efficacy than what could be expected from a purely additive antiviral effect. The potency of this triple combination was next tested in SARS-CoV-2 infected hamsters in a prophylactic setup. To that end, for each of the drugs, intentionally suboptimal or even ineffective doses were selected. Yet, in the lungs of all hamsters that received triple prophylactic therapy (but not in those that received the respective double combinations) no infectious virus was detectable. Our findings indicate that co-administration of approved drugs for the treatment of coronavirus infections should be further explored but also against other families of viruses with epidemic and pandemic potential for which no effective antiviral treatment is available.

## 1. Introduction

Three novel (beta)coronaviruses emerged during the past two decades that are highly pathogenic to humans. These are the severe acute respiratory syndrome coronavirus (SARS-CoV) in 2002, the Middle East respiratory syndrome coronavirus (MERS-CoV) in 2012 and SARS-CoV-2 in 2019. The COVID-19 pandemic, that was caused by SARS-CoV-2, resulted in ~7 million confirmed and ~28 million estimated deaths so far (www.ourworldindata.org/explorers/coronavirus-data-explorer). During and after the emergence of SARS-CoV in 2002, there have been efforts to develop inhibitors of the viral main protease (Mpro or 3CLpro). Twenty years later, this work laid the basis for the rapid development of nirmatrelvir, a potent Mpro inhibitor with pan-coronavirus coverage (Halford, 2022). Remdesivir and molnupiravir are both prodrugs of nucleoside analogues that target, as their 5′-triphosphate metabolite, the viral RNA-dependent RNA polymerase (RdRp) (Li et al., 2023/04) or induce error catastrophe (Kabinger et al., 2021), respectively. These molecules were originally developed for the treatment of infections with other viruses and are endowed with broader spectrum antiviral activity, including activity against coronaviruses. They were therefore repurposed for the treatment of infections with SARS-CoV-2 (Li et al., 2023/04; Toussi et al., 2023). Remdesivir requires intravenous administration, but an oral form, obeldesivir (Mackman et al., 2023), is currently in clinical development for the treatment of COVID-19. GS-441524 is the parent nucleoside of both remdesivir and obeldesivir. Intracellularly, these molecules (GS-441524, remdesivir and obeldesivir) are converted to the tri-phosphate form of GS-441524, which is incorporated in the viral RNA (vRNA) and acts as a chain terminator (Mackman et al., 2023). Molnupiravir, is an oral broad-spectrum anti-viral initially designed to treat infections with alpha- and influenza viruses (Painter et al., 2021) and is in some countries conditionally approved for the treatment of COVID-19^7^. Molnupiravir is intracellularly converted to its 5′-triphosphate form and incorporated in the vRNA leading to lethal mutagenesis and hence inhibition of viral replication (Syed, 2022). Ribavirin is a guanosine analogue with broad-spectrum antiviral activity that has been widely used, in combination with (pegylated)interferon alfa, for the treatment of chronic infections with the hepatitis C virus (HCV), for the treatment of respiratory syncytial virus (RSV) infections (with limited success), and infections with certain viral haemorrhagic fever viruses. For ribavirin there have been different observations regarding its anti-corona virus effect. While several reports describe a lack of activity both *in vitro* and *in vivo* (Al-Tawfiq et al., 2014; Eslami et al., 2020; Hung et al., 2020; Day et al., 2009; Zandi et al., 2020) others did observe some inhibition of SARS-CoV replication (Saijo et al., 2005; Chen et al., 2004). Several mechanisms of action of ribavirin have been proposed, including depletion of intracellular GTP pools, inhibition of viral RNA capping, direct inhibition of the viral RNA polymerase, induction of viral mutagenesis and modulation of host immune responses (for review see (Nyström et al., 2018)).

Multiple combinations of antivirals have already been reported for treatment of SARS-CoV-2 infections (Focosi et al., 2023), but here we explore, for the first time, the potential of a combination of 3 broad-spectrum antiviral nucleosides. The high conservation of viral RdRp, the lack of host homologs and the relative high barrier to resistance make broader spectrum RdRp inhibitors ideal candidates for antiviral therapy. Additionally, combination treatment with antivirals with different mechanisms of action proves to be superior to monotherapy in controlling viral infections caused by the human immunodeficiency virus (HIV) and HCV. Such fixed-dose combinations also suppress or delay the onset of drug-resistance development when compared to single agents (Shyr et al., 2021). The use of combinations of antiviral drugs against SARS-CoV-2, may also result in a more potent effect than monotherapy. In addition, it may allow for dose-reduction and a reduced risk of resistance development. We previously reported that favipiravir significantly potentiates the antiviral efficacy of molnupiravir when co-administered to SARS-CoV-2 infected Syrian hamsters (Abdelnabi et al., 2021a). Similarly, the combined treatment of SARS-CoV-2-infected Syrian hamsters with favipiravir and GS-441524 has been reported to be more efficient compared to the monotherapies (Chiba et al., 2021). Recently, it has also been reported that the combination of suboptimal concentrations of molnupiravir and remdesivir completely blocks the production of SARS-CoV-2 infectious virus particles in the apical wash of human nasal airway epithelium cultures (Jonsdottir et al., 2022). We also demonstrated that the combination of molnupiravir and GS-441524 is highly effective in reducing SARS-CoV-2 replication/infectivity in a hamster infection model (Abdelnabi et al., 2022a). We here explore a next step in these combination studies by exploring the efficacy of the triple combination of GS-441524, molnupiravir and ribavirin against OC43 infection of primary human airway epithelial cells (HAEC) and SARS-CoV2 infections in Syrian hamsters.

## 2. Materials and methods

### 2.1. Virus isolates

Human coronavirus OC43 (HCoV-OC43) was isolated directly on HAE from nasal pharyngeal swabs as previously described (Tapparel et al., 2013). The HCoV-OC43 virus stock was then produced on HAE culture by collecting and pooling apical washes (Boda et al., 2018).

The SARS-CoV-2 variant of concern, B.1.351 (EPI_ISL_896474|2021-01-11 or beta variant) was named according to its own lineage. The strain was isolated from a nasopharyngeal swab of a RT-qPCR confirmed patient. The generation of virus stock was fully reported elsewhere (Abdelnabi et al., 2022b).

All SARS-CoV-2 infectious virus-related work was carried out in a biosafety level 3 (BSL-3) facility at the Rega Institute for Medical Research, KU Leuven, according to institutional guidelines.

### 2.2. Compounds

GS-441524 and ribavirin were purchased from Carbosynth (United Kingdom) while molnupiravir (EIDD-2801) was purchased from Excenen Pharmatech Co. Ltd. (China). In *ex-vivo* experiments, 10 mM stock solution of each compound was prepared in analytical grade dimethyl sulfoxide (DMSO, Sigma). For *in vivo* study, GS-441524 was formulated as a 15 mg/mL stock in a vehicle containing 30% PEG-400 (Sigma) and 1% DMSO in PBS. Molnupiravir was formulated as 50 mg/mL stock in 10% PEG-400 and 2.5% Kolliphor-EL (Sigma) in water. Ribavirin was formulated as 15 mg/ml solution in PBS.

### 2.3. Viral infection of human airway epithelial cells

Nasal human airway epithelial cells (HAEC or MucilAir, cat. no. EP01MD) from a pool of fourteen different donors were obtained from Epithelix (Switzerland) in an air-liquid interphase (ALI) setting and cultured at 37 °C under standard conditions (5% CO2, 100% humidity). Through the course of the experiment itself the cultures were maintained at 34 °C. Prior to infection with HCoV-OC43 (3 × 10^5^ viral copies per insert), the apical site of the cultures were washed once with pre-warmed MucilAir medium (Epithelix, cat. no. EP04MM) and the inserts were then transferred to medium with or without compound. After 1 h pre-incubation, 100 μL of HCoV-OC43 was added at the apical site, incubated for 1.5 h, and the unbound virus was then removed by washing the apical site three times with pre-warmed basal medium. The last of these apical washes was used for quantification of virus background on day 0. Also on day 1, 2, 3, 6, 8, and 10, an apical wash was collected for virus quantification. On day 2 and 4, the basolateral medium of the HAEC culture was refreshed with medium containing compound (or without compound for the controls). On day 6 and 8, the basolateral medium was refreshed with medium without compound for all conditions. Collecting washes was continued for four days after the treatment regimen. Samples were stored at −80 °C until analysis.

### 2.4. SARS-CoV-2-hamster infection model

The hamster infection model of SARS-CoV-2 has been described before (Abdelnabi et al., 2022b). Female Syrian hamsters (*Mesocricetus auratus*, Janvier Laboratories) were housed as pairs in individually ventilated isolator cages (IsoCage N Bio-containment System, Tecniplast) at 21 °C, 55% humidity, and 12:12 day/night cycles. Housing conditions and experimental procedures were approved by the ethics committee of animal experimentation of KU Leuven (license P065–2020). For infection, female hamsters of 6–8 weeks old were first anesthetized with ketamine/xylazine/atropine and inoculated intranasally with 50 μL containing 1 × 10^4^ TCID_50_ SARS-CoV-2 beta variant (day 0). After four days of infection, animals were euthanized for collection of the lungs and further analysis by intraperitoneal (i.p.) injection of 500 μL Dolethal (200 mg/mL sodium pentobarbital).

The doses of compounds were selected such that they result, as monotherapy, in a limited antiviral activity. The selection of the molnupiravir dose (75 mg/kg, BID) was based on a dose-response study against the wild-type strain that we published previously (Abdelnabi et al., 2021b). For GS-441524, we previously tested both 25 and 50 mg/kg BID in our model. The 25 mg/kg BID dose did not result in any reduction in infectious viral loads (unpublished data) while the 50 mg/kg BID dose reduced viral RNA and infectious virus titers in the lung by 1.2 log_10_ copies/mg and 0.5 log_10_ TCID_50_/mg lung tissue, respectively (Abdelnabi et al., 2022a). Therefore we selected GS-441524 at 25 mg/kg BID for the current study. For ribavirin, we explored 25 and 50 mg/kg BID in a pilot study. Both resulted in no antiviral efficacy. However, the 50 mg/kg BID dose resulted in weight loss in the treated hamsters, therefore we selected the 25 mg/kg BID for the current study. Hamsters were treated BID via oral gavage with either vehicle (n = 12), molnupiravir 75 mg/kg (n = 10), GS-441524 25 mg/kg (n = 10), ribavirin 25 mg/kg (n = 10) or combination of the three compounds at the same doses as selected for monotherapy (n = 12) starting from the time of infection (d0) with SARS-CoV-2 beta variant. Smaller groups of animals were also treated with the double combination of either molnupiravir + GS-441524 (n = 6), molnupiravir + ribavirin (n = 4) or GS-441524+ribavirin (n = 4). The BID treatments were done 8 h apart and the compounds were given sequentially for the combination therapy. All the treatments continued until day 3 post-infection (p.i.). Hamsters were monitored for appearance, behavior and weight. At day 4 p.i., hamsters were euthanized as mentioned earlier. Lungs were collected for viral loads quantification. Two independent experiments were done.

### 2.5. End-point titration assay

Hamster’s lung tissues were homogenized using bead disruption (Precellys) in minimal essential medium (MEM) and centrifuged (10000 rpm, 5 min, 4 °C) to pellet the cell debris. Serial dilutions of homogenized supernatant were performed on confluent Vero-E6 cells in 96-well plates to quantify infectious SARS-CoV-2 particles based on the absence or presence of virus-induced cytopathic effect (CPE). Viral titers were calculated by the Reed and Muench method (REED and MUENCH, 1938) and expressed as TCID50 per milligram of tissue.

### 2.6. RNA extraction and quantitative reverse transcription-PCR (RT-qPCR)

Viral RNA of HCoV-OC43 from the apical washes were extracted using QIAamp viral RNA kit (Qiagen, cat. no. 52906) according to the manufacturer’s instructions. Viral RNA was quantified by RT-qPCR using QuantiTect Probe RT-PCR (Qiagen, cat. no. 204445) as previously reported (Boda et al., 2018). The lower limit of quantification (LLOQ) was determined from the standard curve.

### 2.7. Trans-epithelial electrical resistance (TEER)

The electrical resistance of a cellular monolayer is an indicator of the barrier integrity, measured by an epithelial voltohmmeter (EVOM3, World Precision Instruments). During the washing step, 250 μL fresh medium was added to the apical side of the HAE culture, incubated for 15 min at 34 °C, followed by the subsequent resistance measurement according to the manufacturer’s instructions. Briefly, the two electrodes of the “chopstick” were allocated in the apical and basal compartments. The resistance of the culture tissue was then subtracted (100Ω) from the recorded value. The subtracted value (100Ω) is the resistance of the semipermeable membrane without the cell layer, provided by manufacturer. TEER values were presented in units of $\text{Ω}.{\text{cm}}^{2}$ and calculated as:

$$TEER\left(\text{Ω}.cm^{2}\right)=Resistance\left(\text{Ω}\right)\times Area\left(cm^{2}\right)$$

where Resistance and Area are the cell specific resistance and the surface of semipermeable membrane, respectively. The TEER within one specific day was normalized to the uninfected-untreated controls.

### 2.8. Cytotoxicity measurement

Compound/Virus-induced toxicity was measured by a colorimetric assay to detect lactate dehydrogenase (LDH) released into the basolateral medium using CyQUANT LDH Cytotoxicity Assay kit (Invitrogen, cat. no. C20300). In short, 50 μL of each sample basal medium was transferred into a 96-well plate and mixed with 50 μL of reaction mixture. Samples were incubated at room temperature for 30 min, protected from light, followed by the addition of 50 μL of stop solution to each well. Medium from HAE cultures that were lysed by Triton X-100 10% overnight was used as maximal LDH activity control. In parallel, the fresh MucilAir medium served as spontaneous LDH activity control and LDH positive control were included in each experiment. The absorbance at 490 nm and 680 nm were measured. The 680-nm absorbance values were subtracted from 490-nm absorbance values to calculate the LDH activity. Percentage (%) cytotoxicity was determined according to following formula:

$$\%\;cytotoxicity=\left(\frac{\text{Sample}\;\text{LDH}\;\text{activity}-\text{Medium}\;\text{LDH}\;\text{activity}}{\text{Maximal}\;\text{LDH}\;\text{activity}-\text{Medium}\;\text{LDH}\;\text{activity}}\right)\;\;\;\;\;\;\;\;\;\;\;\;\;\;\;\;\times 100$$

### 2.9. Pharmacodynamic interaction analysis

The interactions within double and triple therapies (ribavirin + GS-441524, molnupiravir + GS-441524, ribavirin + molnupiravir, and ribavirin + GS-441524 + molnupiravir) were analysed with the Bliss independence zero-interaction theory. According to this pharmacodynamic theory, if three molecules (A, B, and C) do not interact, i.e. they act independently, then the effect E of their combined actions can be predicted using the probability law of independent events (Poch et al., 1990). This predicted effect $E_{ABC,Pred.}$ is determined by the formula:

$$E_{ABC,Pred.}=1-\prod \left(1-E\right)$$

where $\prod \left(1-E\right)$ is the product of the observed effects of each single agent alone (i.e. for a triple combination $E_{ABC,Pred.}=1-\left(1-E_{A}\right)\times \left(1-E_{B}\right)\times \left(1-E_{C}\right)$). Of note, the effect of viral inhibition/reduction in the latter equation can be converted from log_10_ viral reduction:

$$E=1-10^{-L}$$

where E is the inhibition/reduction and L is the log_10_ viral reduction.

The observed combined effect $E_{ABC,Obs.}$ is then compared with $E_{ABC,Pred.}$. If $E_{ABC,Obs.}>E_{ABC,Pred.}$, the compounds act in a synergistic manner; if $E_{ABC,Obs.}<E_{ABC,Pred.}$, there is antagonism whereas $E_{ABC,Obs.}=E_{ABC,Pred.}$, indicates additivity.

### 2.10. Statistical analysis

All statistical comparisons in the study were performed in GraphPad Prism 9 (GraphPad Software, Inc.). Statistical significance was determined using the ordinary one-way ANOVA with Dunnett’s multiple comparison test (*ex vivo* data) or the non-parametric Mann-Whitney *U* test (*in vivo* data). P-values of <0.05 were considered statistically significant. In the figures, accurate p-value was indicated for each group in comparison with the control/vehicle group.

## 3. Results

### 3.1. Antiviral effect of triple combination of GS-441524, molnupiravir and ribavirin in HNAEC cultures infected with HCoV-OC43

**Fig. 1.**
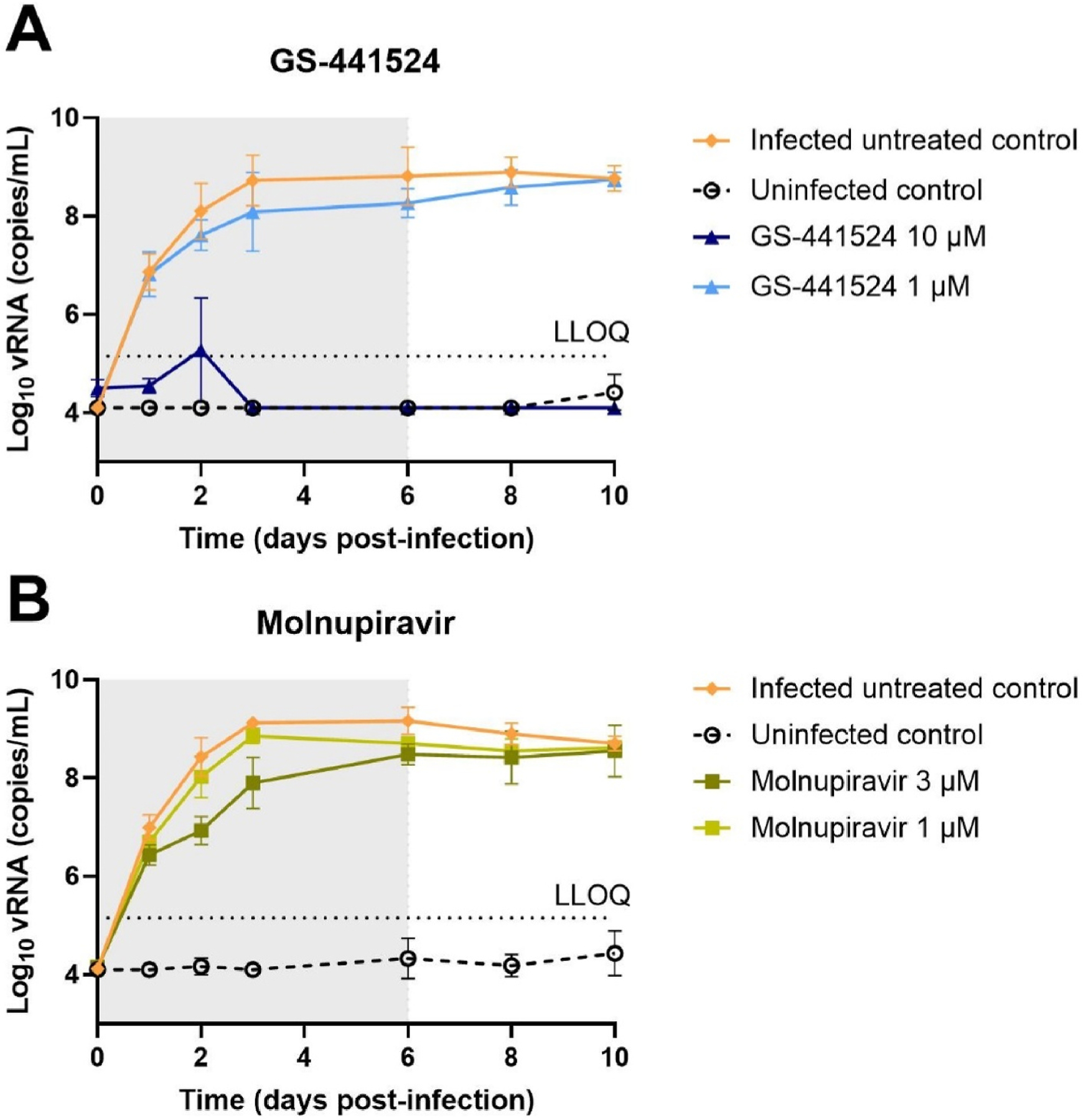
Antiviral activity of GS-441524 and molnupiravir against HCoV-OC43 in HAEC cultures. Compounds were added to the basal medium at different concentrations 1 h prior to infection with HCoV-OC43 at 34 °C. Basal medium, with or without compounds, was refreshed every other day from day 0 to day 6. Viral RNA in apical washes were quantified by RT-qPCR. Dose-response and time-dependent activity of GS-441524 (A) and molnupiravir (B). All data are mean ± SD of two independent experiments with each 2 technical replicates for the uninfected control and 3 technical replicates for the other conditions. Grey box indicates time of treatment. LLOQ represents lower limit of quantification.

**Fig. 2.**
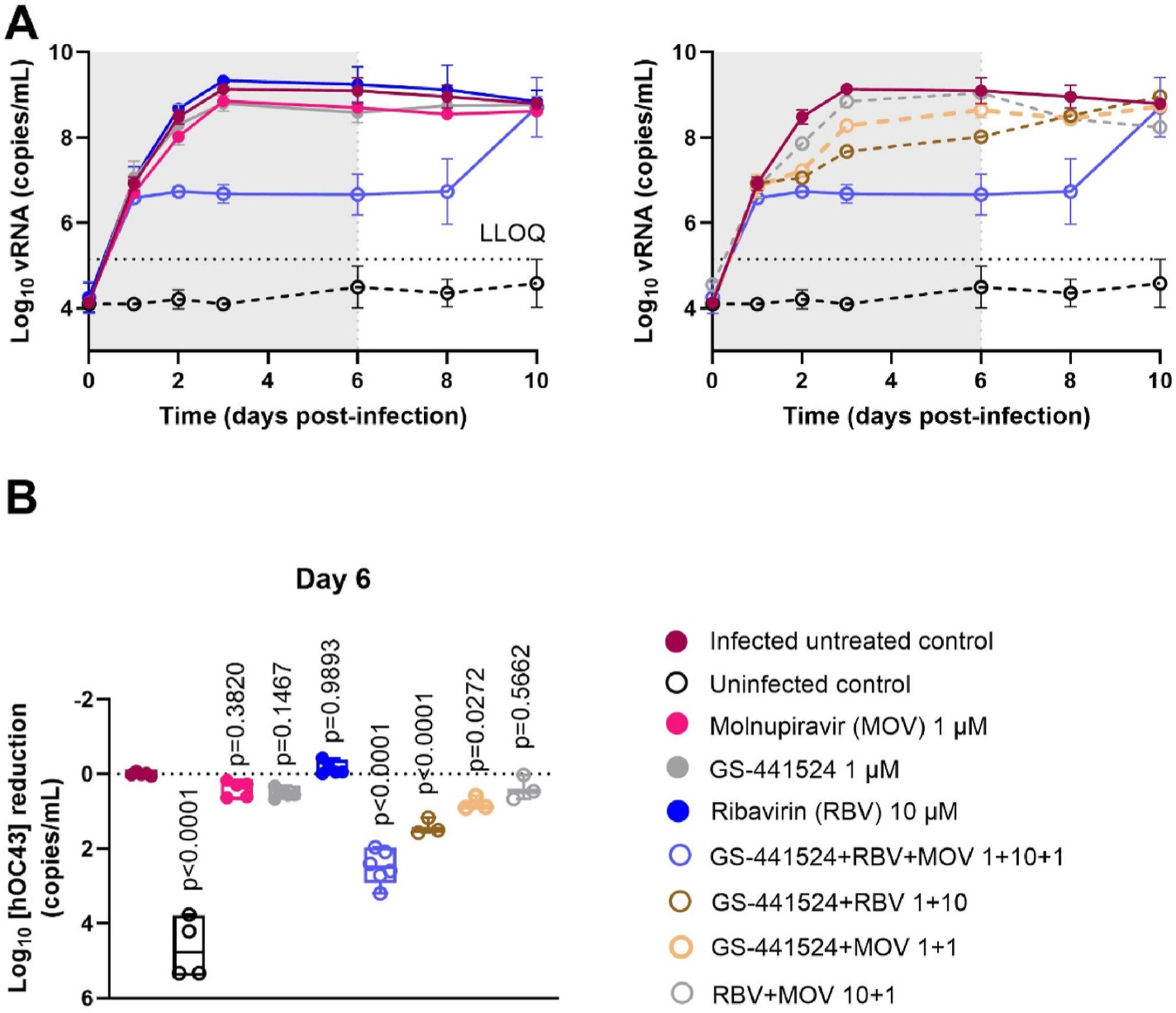
Comparing the antiviral activity of combinations of GS-441524, molnupiravir (MOV), and ribavirin (RBV) on HCoV-OC43 replication in nasal human airway epithelial cell (HAEC) cultures. Compounds were added to the basal medium starting 1 h before infection and treatment continued for 6 days. Nasal HAEC were infected with HCoV-OC43 at 3 × 10^5^ copies/insert and incubated at 34 °C. Viral RNA in apical washes was quantified by RT-qPCR. (A) Kinetics of HCoV-OC43 replication with or without different therapies. Left monotherapies and triple therapy and right dual therapies and triple therapy. (B) Statistical analysis of the antiviral activities of each treatment on day 6 p.i. The double combinations were tested in one experiment with 3 technical replicates. The other conditions were tested in two independent experiments with each 2 technical replicates for the uninfected control and 3 technical replicates for the other conditions. LLOQ represents the lower limit of quantification. Statistical significance between infected untreated control and other groups was calculated by one-way ANOVA with two-sided Dunn’s post hoc test. Data are mean ± SD of at least three biological replicates.

To explore the antiviral efficacy of combinations of two or three drugs [GS-441524 (the parent nucleoside of remdesivir), molnupiravir and ribavirin] HAEC cultures were infected with HCoV-OC43. The kinetics of virus replication was monitored over several days through quantification of virus released at the apical site of the cultures. We first defined the effective and suboptimal concentration of GS-441524 and molnupiravir for inhibition of HCoV-OC43 replication in nasal HAEC cultures. Treatment with 10 μM GS-441524 results in a “cure” of the cultures since no viral rebound is observed when the therapy is stopped. At 1 μM of GS-441524, however, the viral load is only marginally reduced (Fig. 1A). At 3 μM, molnupiravir treatment results in a significant inhibition of viral replication (up to 1.5 log_10_ vRNA reduction) whereas 1 μM is clearly suboptimal (Fig. 1B). Based on these results, we selected for both GS-441524 and molnupiravir a concentration of 1 μM as suboptimal for subsequent combination experiments in nasal HAEC cultures. Ribavirin, at 10 μM, has no measurable effect on HCoV-OC43 replication in HAEC cultures (Fig. 2). Next, the effect of 1 μM GS-441524, 1 μM molnupiravir and 10 μM ribavirin, either alone or in double or triple combinations on HCoV-OC43 replication was assessed (Fig. 2A). Statistical analysis was performed for the results on day 6 p.i., which corresponds with the last day of treatment (Fig. 2B). Neither the monotherapies nor the double combinations of either GS-441524 plus molnupiravir or molnupiravir plus ribavirin has a significant effect on virus replication. The combinations GS-441524 plus ribavirin results in a 1.4 log_10_ reduction in viral RNA but when combined with molnupiravir (thus GS-441524 plus ribavirin plus molnupiravir), 2.5 log_10_ RNA reduction in viral RNA is achieved. Upon cessation of therapy, virus replication increases again for both conditions.

Since the monotherapies of 1 μM GS-441524, 1 μM molnupiravir and 10 μM ribavirin do not show a measurable antiviral effect, it is not possible to calculate the expected additive effect of the combination based on the Bliss independence zero-interaction theory (described in the methods). Therefore we use an alternative approach in which we calculate a maximal expected additive effect based on an estimated maximal efficacy of the monotherapies. From the results shown in Fig. 2B it is clear that the monotherapies have an effect of less than 0.7 log_10_ vRNA reduction (which corresponds with an effect of 80% inhibition), so we can argue that the maximal effect of each monotherapy is less than 80%. The maximal expected predicted additive inhibition of the triple combination, according to the Bliss model, is therefore 1 - (1–80%) × (1–80%) ×(1–80%) = 99.2% which corresponds with 2.1 log_10_ vRNA reduction. If we compare this with the observed inhibition of 2.5 log_10_ vRNA reduction (which corresponds with 99.7% effect with interquartile range of 99.3%–99.8%), we conclude that the observed inhibition of the triple combination is significantly higher than the maximal expected additive effect. Thus the antiviral effect of the triple combination is synergistic. Of note, there was no indication of toxicity in any of the conditions tested in trans-epithelial electrical resistance (TEER) measurement (i.e. determining the integrity and permeability of lung tissue) or when quantifying levels of released lactate dehydrogenase enzyme (Supplementary data Fig. S1).

### 3.2. The combined treatment of suboptimal doses of GS-441524 and molnupiravir with ribavirin efficiently inhibits SARS-CoV-2 replication in hamsters

**Fig. 3.**
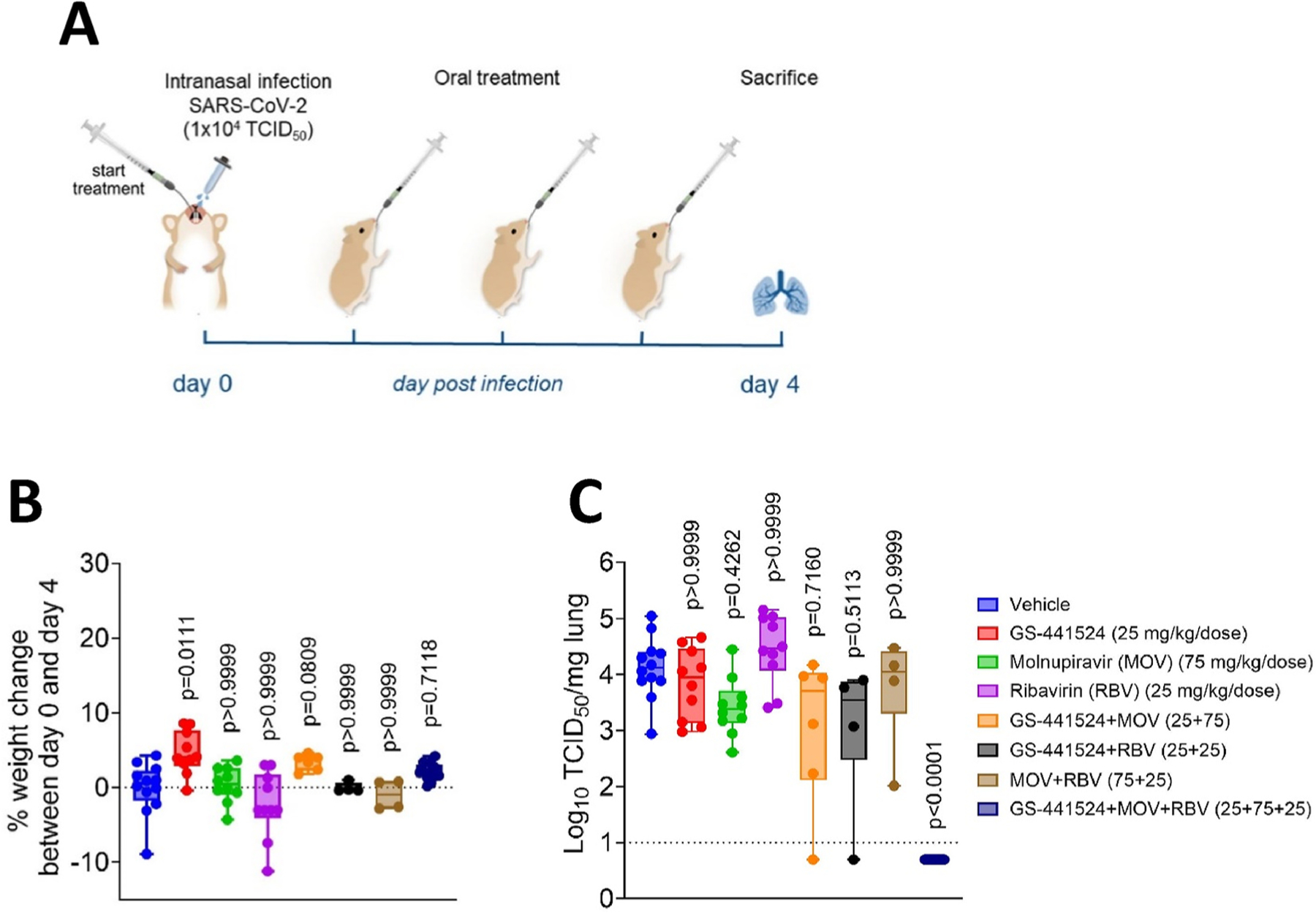
Antiviral effect of combinations of GS-441524, molnupiravir (MOV), and ribavirin (RBV) on SARS-CoV-2 infection in hamsters (A) Design of the study. (B, C) Box plots, with whiskers indicating min to max, representing body weight change, and infectious viral titers in the lungs. (B) Weight change shown as percentage of change on total weight between day 0 and day 4 p.i. (C) Infectious virus titers in the lungs at day 4 p.i. as determined by end-point titration and shown as log_10_ TCID_50_ per mg lung tissue. The lower limit of quantification of the titration is indicated with the dotted line. Statistical analysis was performed with the Kruskal-Wallis test with Dunn’s comparison. Data are pool of three independent experiments with n = 12 for vehicle and the triple therapy-treated group, n = 10 for the monotherapy-treated groups and n = 4 or 6 for the dual therapy groups.

We next studied whether the triple combination of GS-441524, molnupiravir and ribavirin results also in SARS-CoV-2 infected hamsters in a more than additive activity. Female hamsters were intranasally infected with 1 × 10^4^ TCID_50_ of SARS-CoV-2 (beta variant, lineage B.1.351) and were orally treated twice a day (BID) for four consecutive days starting 1 h before infection. Doses of each of the drugs were in preliminary studies (see methods) determined to be suboptimal to inactive. In the current experiment, GS-441524 alone at 25 mg/kg/dose results in 0.2 log_10_ reduction of infectious virus titers in the lungs (at day 4 post infection), molnupiravir at 75 mg/kg/dose in 0.7 log_10_ reduction and ribavirin at 25 mg/kg/dose in no observable reduction of viral titers. None of the changes in infectious virus titers by the monotherapies is statistically significant. On day 4 p.i., infected hamsters from all groups exhibited <10% weight change indicating that all treatments were well tolerated (Fig. 3). The combination of GS-441524 with molnupiravir reduced infectious titers in the lungs by 0.6 log_10_ (p = 0.72), compared to the vehicle control. No statistically significant reduction in infectious titers in the lungs was observed in the ribavirin plus molnupiravir group. The combination GS-441524 plus ribavirin resulted in 0.80 log_10_ (p = 0.51) reduction in infectious titers. Finally, in all 12 animals that had been treated with GS-441524 plus molnupiravir plus ribavirin, infectious titers in the lungs were reduced by > 3.7 log_10_ (p < 0.0001) to undetectable levels (Fig. 3).

Given that the results of mono- and dual therapies show no statistically significant reduction in viral load (p > 0.05) we need to be prudent with a pharmacodynamic interpretation. The viral load reductions of the monotherapies correspond with an effect of 37% for GS-441524, 80% for molnupiravir and 0% for ribavirin. Based on the Bliss independence zero-interaction theory (see methods) this would predict an 87% additive effect for the triple combination which corresponds to a 0.90 log_10_ reduction in infectious titers. As we observe a >3.7 log_10_ reduction there is a clear indication of synergy.

## 4. Discussion

Remdesivir and molnupiravir, both prodrugs of nucleoside analogues, were initially developed for other indications (treatment of Ebola and influenza infections, respectively) but were during the SARS-CoV-2 pandemic “repurposed” for the treatment of COVID-19. Besides these nucleoside analogues, potent coronavirus main protease (3CLpro) inhibitors have been developed (nirmatrelvir, ensitrelvir) or are in development. So far, fortunately, the development of resistance of SARS-CoV-2 to the above-mentioned drugs has been limited. However, in such case that during future outbreaks with (a) novel coronavirus(es), such antivirals would be more widely used (in particular during the first phases of a pandemic when vaccines are not yet available), drug resistance may become an issue. Combining drugs with a different resistance profile may then delay or avoid the emergence of such variants. Hence, we believe that it might be prudent to explore the combination of existing drugs against coronaviruses in general and SARS-CoV-2 in particular. As a first step, we aimed to explore the antiviral potency of some combinations and then in a next stage (but not part of this study) explore whether such combinations also prevent the potential development of resistance. To that end, we studied the combination of GS-441524 (parent nucleoside of remdesivir) and molnupiravir in addition with ribavirin (which is not known as a coronavirus inhibitor) whether ribavirin may further modulate the combined activity of these molecules. The latter was inspired by the fact that ribavirin, as monotherapy, is not active in the treatment of chronic HCV infections; yet markedly potentiates the antiviral efficacy of (pegylated)interferon alpha without being effective on its own (Dixit et al., 2004/12). Thus, ribavirin is able to “modulate” the antiviral activity of interferon, yet via an unknown mechanism. Furthermore, these three molecules were selected because they each exert activity against families of viruses beyond the coronaviruses (such as para- and orthomyxoviruses); hence the hope that the data from this study may be relevant for the setup of similar studies with other viruses.

We first explored the antiviral activity of triple therapy in nasal HAEC cultures grown at the air liquid interface infected with HCoV-OC43. Concentrations of each of the drugs were selected such that they had alone only a minimal effect on viral replication. The triple combination of each of these drugs used at suboptimal concentrations, resulted in a pronounced antiviral effect, whereas the double combinations did not. This synergy may be explained by the complementary mechanisms of antiviral action of these molecules. First, ribavirin 5′-monophosphate (RMP - an intracellular metabolite of ribavirin) acts as a competitive inhibitor of inosine 5′-monophosphate dehydrogenase (IMPDH), leading to the depletion of (intracellular) guanosine (deoxy) nucleoside triphosphate (GTP) pools. Since GTP is an essential building block for viral RNA synthesis, this consequently results in inhibition of the replication of RNA viruses (Paeshuyse et al., 2011). Decrease in GTP levels limits the concentration of ATP pools and *vice versa* (Pelley and Pelley, 2012), facilitating the incorporation of other purine nucleoside analogues into nascent RNA such as GS-441524 (an adenosine analogue) which acts as a chain terminator. As a result, ribavirin may increase the activity of GS-441524 that ultimately prevents RNA strand elongation and viral replication. Similar synergistic interactions between ribavirin and other purine nucleoside analogues have been reported in Lassa virus and HIV infections (Margot and Miller, 2005; Oestereich et al., 2016; Hartman et al., 1991). Second, the active intracellular molnupiravir metabolite, β-D-N4-hydroxycytidine-triphosphate (NHC-TP), is incorporated into new synthesized viral RNA strands to replace either CTP or UTP, thereby causing C-to-U and G-to-A transition mutations which results in error catastrophe and lethal mutagenesis (Syed, 2022; Urakova et al., 2018). Therefore, the combined actions of error induction (molnupiravir), chain termination (GS-441524) with depletion of GTP levels (ribavirin) (also the combination of ribavirin and GS-441524) may explain the efficacious suppressing of coronavirus replication.

Inspired by the observations with HCoV-OC43 in nasal HAEC cultures, we next explored whether the triple combinations of the three drugs, each used at suboptimal doses, would also result in a pronounced antiviral activity in the SARS-CoV-2 hamster infection model in a prophylactic setup. Whereas each of the drugs alone reduced only minimally (GS-441524 0.2 log_10_ and molnupiravir 0.7 log_10_) or not (ribavirin) the infectious viral titers in the lungs; when they were combined, no infectious virus was detectable in the lungs of any of the 12 treated hamsters (>3.7 log_10_ reduction). A quantitative analysis (see result section) shows that the additive Bliss model predicts only a 0.9 log_10_ reduction for the triple combination when starting from the results of the monotherapies. Since the observed viral load reduction is significantly higher (>3.7 log_10_ reduction) we conclude that the triple combination has a synergistic effect. The modest antiviral effect of molnupiravir alone (at 75 mg/kg/dose) is consistent with data from other studies in hamsters (Abdelnabi et al., 2021b; Jeong et al., 2022; Sheahan et al., 2020). This inhibition is associated with an increased mutation frequency (Abdelnabi et al., 2021b; Sheahan et al., 2020). The 75 mg/kg molnupiravir dose is equivalent to 10 mg/kg of the Human Equivalent Dose (i.e. 600 mg) when normalized to the human body surface area (Reagan-Shaw et al., 2008). This is less than the standard dosing regimen of molnupiravir in outpatients with COVID-19 (800 mg, BID) (Jayk Bernal et al., 2022). Also, no statistically significant antiviral potency was observed when hamsters were treated with 25 mg/kg/dose GS-441524, which is in agreement with a previous report (Abdelnabi et al., 2022a). For ribavirin there have been different observations regarding its anti-corona virus effect. While several reports describe a lack of activity both *in vitro* and *in vivo* (Al-Tawfiq et al., 2014; Eslami et al., 2020; Hung et al., 2020; Day et al., 2009; Zandi et al., 2020) others did observe some inhibition of SARS-CoV replication (Saijo et al., 2005; Chen et al., 2004). A possible explanation is the excision of ribavirin-5′monophosphate after incorporation by the coronavirus exoribonuclease (Ferron et al., 2018). In our hamster study we used a dose of 25 mg/kg BID ribavirin which is equivalent to 200 mg BID Human Equivalent Dose (Reagan-Shaw et al., 2008). This is less than the standard dose of 800 mg/day in the treatment of HCV infections. If our finding could be translated to the human scenario, it is presumable that the triple therapy results in a potent antiviral effect at clinically readily achievable doses of remdesivir, molnupiravir and ribavirin with possibly a lower probability of associated adverse events. An important aspect, and still to be explored, is whether this triple combination may also prevent the development of possible drug-resistant variants. In addition we want to point out that we have limited our studies to a prophylactic setup to demonstrate the proof-of-concept. Experiments with delayed initiation of therapy are needed to explore any potential clinical utility.

In conclusion, we demonstrate that when suboptimal to even almost inactive doses of the nucleoside analogues GS-441524, molnupiravir and ribavirin are combined, this results in a pronounced prophylactic antiviral potency which is most remarkable in a SARS-CoV-2 hamster infection model. Since these and some other small molecule antivirals exert also antiviral activity against viruses belonging to other families, it may, in the context of epidemic and pandemic preparedness, be important to explore whether the triple combination studied here, or yet other combinations, may hold promise for the treatment of life-threatening viral infections. Also, for those viral infections for which there is today no treatment available, it may be explored whether certain combinations hold promise.

## Supplementary Material

1

## Data Availability

Data will be made available on request.
